# Landscape- and local-scale habitat influences on occurrence and detection probability of Clark’s nutcrackers: Implications for conservation

**DOI:** 10.1371/journal.pone.0233726

**Published:** 2020-05-29

**Authors:** Taza D. Schaming, Chris S. Sutherland

**Affiliations:** 1 Northern Rockies Conservation Cooperative, Jackson, Wyoming, United States of America; 2 Department of Environmental Conservation, University of Massachusetts Amherst, Amherst, Massachusetts, United States of America; USDA Forest Service, UNITED STATES

## Abstract

Whitebark pine (*Pinus albicaulis*), a keystone species and an obligate mutualist of the Clark’s nutcracker (*Nucifraga columbiana*), is rapidly declining throughout its range. Evidence suggests this decline is leading to a downward trend in local nutcracker populations, which would in-turn decrease whitebark pine regeneration. Our objectives were to (1) evaluate temporal variation in nutcracker habitat use as a function of whitebark pine and Douglas-fir (*Pseudotsuga menziesii*) habitat, at local and landscape scales, (2) develop metrics for predicting when whitebark pine communities require intervention to sustain nutcracker visitation, and (3) test McKinney et al. (2009) and Barringer et al.’s (2012) models predicting nutcracker occurrence. Between 2009 and 2013, we carried out 3,135 audio-visual Clark’s nutcracker surveys at 238 random points in the southern Greater Yellowstone Ecosystem. Using Bayesian occupancy models and cross-product model selection, we evaluated the association between nutcracker occurrence and habitat variables during five stages of the nutcracker annual cycle, while accounting for imperfect detection. Nutcracker occurrence was most strongly associated with the presence of cone-bearing whitebark pine trees (rather than cone density) and the area of whitebark pine on the landscape. To promote a high, >75%, probability of occurrence at a site within the study area, we recommend a management plan that achieves a landscape composed of a minimum of 12,500–25,000 ha of cone-bearing whitebark pine habitat within a 32.6 km radius. Additionally, an optimal habitat mosaic includes moderate levels of Douglas-fir habitat. Models currently used to guide whitebark pine management strategies underpredicted nutcracker occurrence in our study area, suggesting these strategies may not be appropriate in the region. We cannot predict how this mutualistic relationship will change as the population density of each species shifts. We therefore suggest conducting periodic surveys to re-evaluate the relationship as the environment changes and management strategies are implemented.

## Introduction

Species declines can have significant, detrimental effects on forest community dynamics and persistence [1–3]. To assess a mutualism’s stability and resilience when the population of one of the partners begins to decline, it is critical to understand environmental factors which impact the species’ interactions. Whitebark pine (*Pinus albicaulis*), a keystone and foundation species, is a critical component of alpine and subalpine forest ecosystems in the western United States, contributing to biodiversity, ecosystem structure and hydrologic cycling [4–6]. Many animal species depend on its high-fat, high-energy nuts [4,7]. Whitebark pine is rapidly disappearing range-wide due to widespread infection by non-native white pine blister rust (*Cronartium ribicola*), outbreaks of mountain pine beetles (*Dendroctonus ponderosae*), decades of fire suppression, and the interaction of these factors with climate change [4,8,9]. Due to ongoing threats and heavy mortality, whitebark pine is a candidate for listing under the U.S. Endangered Species Act, and is listed as an endangered species in Canada [10,11]. The high whitebark pine mortality may seriously reduce biodiversity and disrupt many species interactions [12], including its own relationship with Clark’s nutcrackers (*Nucifraga columbiana*). Whitebark pine is dependent on Clark’s nutcrackers because its seeds sprout almost exclusively from nutcracker seed caches [13,14]. Because of this obligate dependence, information on nutcracker habitat use is important to informing whitebark pine management strategies.

Clark’s nutcrackers are important for forest regeneration because they disperse seeds of at least ten species of conifers in the western U.S. [See 15]. Nutcrackers shape the ecosystem by annually storing an estimated 32,000 to 98,000 seeds in thousands of separate locations; seeds not retrieved for food are able to germinate [13,16]. They rapidly and effectively move seeds up to 32.6 km, across latitudinal and elevational gradients, and into disturbed areas and newly available habitats [4,17]. The continued association between Clark’s nutcrackers and conifers may be important in maintaining and managing many forests in the western U.S.

Evidence suggests that Clark’s nutcracker populations are declining in large parts of their range, likely as a result of the widespread decline of whitebark and limber pines (*Pinus flexilis*) [4,18,19]. The birds are still relatively common in the Greater Yellowstone Ecosystem, despite the high mortality of whitebark pines in the region [20,21]. Nutcrackers do forage on seeds of numerous conifers, including three species in the study area: whitebark pine, limber pine, and Douglas-fir (*Pseudotsuga menziesii*). Limber pines are few and patchy in the area, but Douglas-fir is an important alternative seed source in the region [22,23]. We therefore predicted that Clark’s nutcracker occurrence (use) patterns would be primarily influenced by both whitebark pine and Douglas-fir habitat.

Extensive whitebark pine restoration efforts are underway, assuming that once a certain level of whitebark pine restoration has been achieved, Clark’s nutcrackers will be available to continue dispersing seeds [24,25]. Recent research shows a significant, positive relationship between whitebark pine seed source health, and nutcracker-dispersed seedling density in adjacent burns [26]. Both McKinney et al. [18] and Barringer et al. [19] suggested that a threshold of 1,000 cones/ha, on average, is necessary for a high likelihood of nutcracker presence and hence potential seed dispersal, and they determined that this level of cone production can be met by live whitebark pine basal areas of >5.0 m^2^/ha or ≥2.0m^2^/ha, respectively. Forest managers have since used these thresholds when developing whitebark pine management strategies [25,27]. However, these models need to be tested in additional regions to assess whether the current whitebark pine restoration guidelines are accurate in diverse locations. Additionally, as nutcracker habitat use occurs at a landscape scale, more detailed investigation is needed to assess the local spatial extent over which habitat metrics influence nutcracker occurrence [18,19].

The primary goal of this study was to determine whether presence or density of whitebark pine cone crop, presence or density of whitebark pine and Douglas-fir habitat at the local scale, and/or proportion of whitebark pine and Douglas-fir habitat at the landscape scale, best predicted Clark’s nutcracker occurrence. Because Clark’s nutcracker behavior varied considerably throughout the year, we examined occurrence separately for each of five stages of the birds’ annual cycle, and based predictions for each stage on Clark’s nutcracker ecology [21,23,28]. We considered the breeding season the first date in any year a Clark’s nutcracker was seen building a nest (March 5), through the last date a nestling was observed on a nest (June 15) [21]. Early summer was June 16 through the day before we observed nutcrackers eating immature whitebark pine seeds. Late summer was the period when Clark’s nutcrackers were seed predators, eating immature whitebark pine seeds, fall seed harvest was when Clark’s nutcrackers were potential seed dispersers, harvesting and caching mature whitebark pine seeds, and post-harvest was when we observed no mature whitebark pine cones remaining on the trees.

We predicted (1) nutcracker behavior would only be influenced by cones when they were available, during late summer and fall harvest, (2) local presence of whitebark pine could influence nutcracker occurrence during early summer when nutcrackers presumably scouted for future seed sources, and during late summer and fall harvest when the birds were harvesting seeds, (3) the area of whitebark pine on the landscape would influence occurrence throughout the year, since even when the birds were not harvesting seeds, they would be scouting or consuming previously cached seeds, and (4) local presence and area of Douglas-fir on the landscape would impact nutcracker occurrence during breeding season and post-harvest stages. We observed nutcrackers regularly harvesting Douglas-fir seeds late winter into the breeding season, and harvesting and caching Douglas-fir seeds post-whitebark pine harvest; additionally, nutcrackers selected Douglas-fir habitat for their breeding season home range [23]. We predicted detectability would be impacted by tree density as our ability to both see and hear birds may have decreased with higher tree density, and whitebark pine importance value during the fall harvest because, during that time, we observed individual birds calling more loudly and frequently, and moving in larger, noisier groups when foraging on whitebark pine cones.

We assessed variation in inference about occurrence patterns when using alternative (<100, infinite) survey radii. Nutcrackers detected within 100 m were considered to be more closely associated with the survey stand, while nutcrackers observed at an unlimited distance were using the local landscape, but not necessarily the survey stand. Using alternative radii also allowed for closer comparison with McKinney et al. [18] and Barringer et al.’s [19] previous studies, which documented nutcrackers observed within different radii, within 1 ha and an infinite distance, respectively. To inform current management strategies, we determined whether McKinney et al. [18] and Barringer et al.’s [19] previous models accurately predicted nutcracker occurrence data from our study. Finally, because counting whitebark pine cones is labor intensive, we also evaluated if it was possible to predict cone crop at a location based on habitat variables.

## Materials and methods

### Ethics statement

This research was approved by the Cornell University Institutional Animal Care and Use Committee (protocol # 2008–0176). We conducted all field work under U.S. Forest Service Special-Use Authorization # JAC747002 (2009–2013) and Grand Teton National Park Scientific Research and Collecting Permit #’s GRTE-2011-SCI-0052 and GRTE-2012-SCI-0069.

### Field methodology

#### Study area

**Fig 1:**
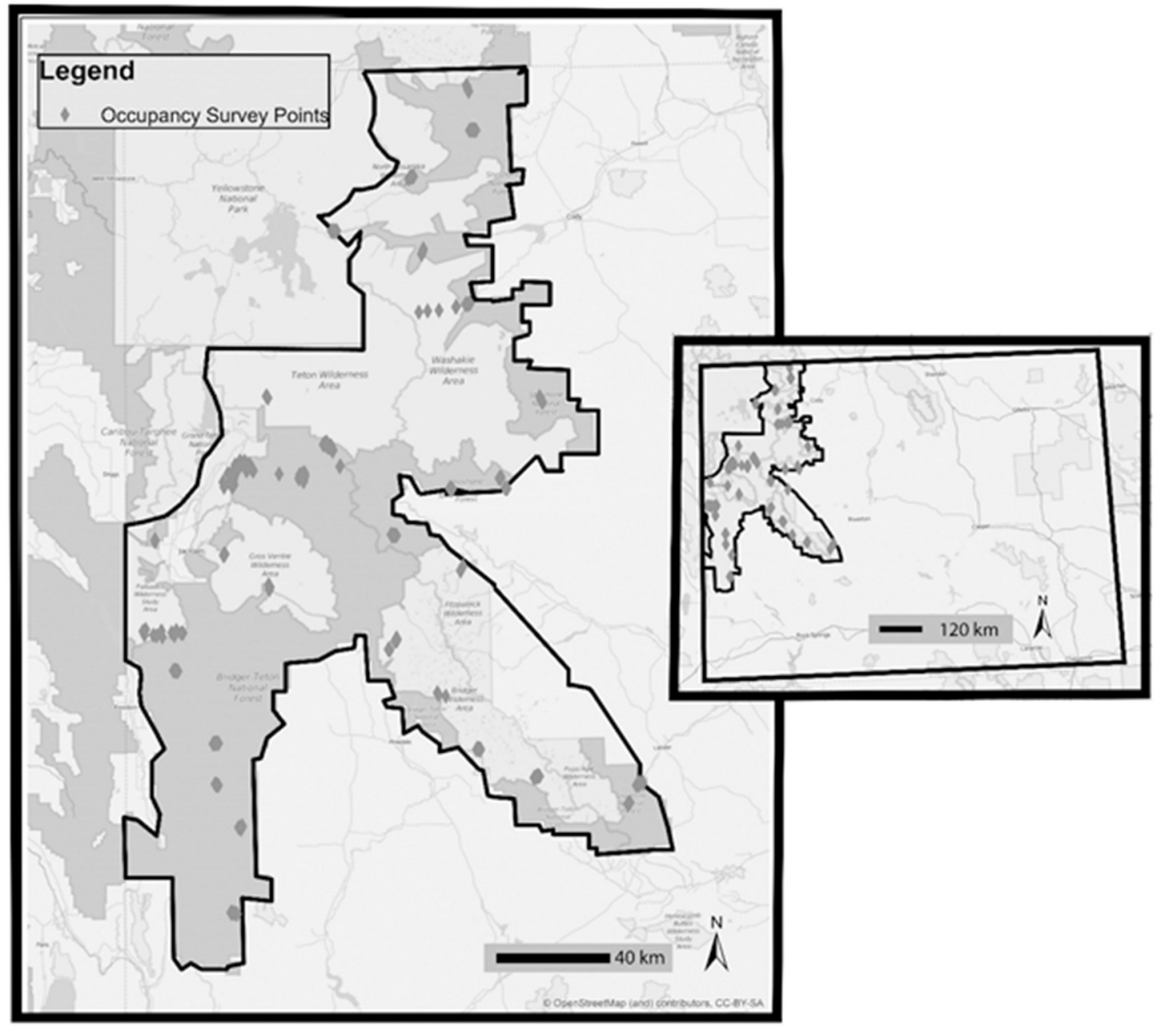
Study area in the Greater Yellowstone Ecosystem. The inset map depicts the study area within the state of Wyoming. (Reprinted from map data © OpenStreetMap contributors, map layer by Esri, under a CC BY-SA 4.0 license).

This study is based on five years (2009–2013) of Clark’s nutcracker surveys carried out in the Greater Yellowstone Ecosystem, in northwestern Wyoming, primarily in Bridger Teton and Shoshone National Forests, and Grand Teton National Park (25,050 km^2^; bounded by 45°00’01” N north, 42°09’14” N south, 111°02’56”W west, and 108°42’55”W east; Fig 1). We conducted 3,135 point surveys at 238 random sites. Sites ranged in elevation from 1,843 to 3,372 m, and were located in a habitat mosaic dominated by six conifer species: whitebark pine, Douglas-fir, limber pine, lodgepole pine (*Pinus contorta*), Engelmann spruce (*Picea englemannii*), and subalpine fir (*Abies lasiocarpa*). The conifer habitat was interspersed with aspen (*Populus tremuloides)*, sagebrush (*Artemesia tridentata*), grassy open areas, high mountain meadows and rocky outcroppings.

#### Clark’s nutcracker surveys

We established 238 random survey points within seven km of whitebark pine habitat. The six dominant conifer species were present at a varying number of points: whitebark pine (49%), limber pine (22%), Douglas-fir (32%), lodgepole pine (38%), Engelmann spruce (47%), and subalpine fir (63%) [21]. For details on point placement, see Schaming [21].

Each year, we conducted surveys at newly established points and a subset of previously established points (Table 1). We eliminated surveys at sites <400 m apart after 2009, and did not revisit some sites due to logistics. One survey consisted of three sequential ten-minute point counts, after an initial three-minute waiting period to minimize disturbance caused by our arrival. Three counts were necessary to evaluate detectability of Clark’s nutcrackers as a function of habitats variables [29,30]. Surveys were conducted when precipitation was absent or limited to light intermittent rain or snow. We recorded whether Clark’s nutcrackers were detected through sight and/or calls, and the distance to each observation.

**Table 1:** Number and dates of surveys during each stage of the annual cycle.

Stage of the annual cycle	Sample size (survey dates)					
	2009	2010	2011	2012	2013	Total
Breeding season	0	0	123 (Mar 15 –Jun 10)	0	261 (May 19 –Jun 15)	384
Early summer	0	0	51 (Jun 16 –Jun 30)	6 (Jun 22)	132 (Jun 16 –Jun 29)	189
Late summer	144 (July 15 –Aug 11)	3 (July 15 –July 28)	189 (July 7 –Aug 3)	6 (July 12 –July 27)	411 (July 12 –Aug 9)	753
Fall seed harvest	144 (Aug 18 –Aug 30)	0	165 (Aug 16 –Sept 15)	72 (Aug 13 –Sept 6)	612 (Aug 12 –Sept 28)	993
Post-harvest	144 (Sept 9 –Sept 22)	0	0	45 (Sept 10 –Sept 27)	18 (Sept 29 –Oct 1)	207

#### Habitat surveys

*Whitebark pine density*. To determine the annual number of cone-bearing whitebark pine trees per hectare, once whitebark pine cones were visible, we conducted one set of belt transects, rectangular sampling plots, at each survey point. One set consisted of four separate 10 x 50 m transects, extending from the point in each cardinal direction, for a total of 1,900 m^2^ (the first 5 m of the western and eastern transects were discarded due to overlap). Within each transect, we counted the number of dead, cone-bearing, and non-cone-bearing whitebark pine trees >7 cm DBH (diameter at breast height; 1.4 m). All trees with green foliage were classified as ‘‘live”, regardless of condition.

*Cone density*. At each survey point, once cones were visible, we randomly chose four cone-bearing whitebark pine trees. We followed a randomly selected azimuth bearing until locating a cone-bearing whitebark pine within 50 m. If four cone-bearing whitebark pine trees were not available within 50 m, we counted cones on as many as were available. Whitebark pine trees regularly grow in clusters; we considered each stem that split from a clump below 1.4 m to be a separate tree. Each year, we counted the number of cones on the same four trees. In 2009, we counted cones twice at each point, July 12–31, then August 21–30; in 2010–2013, we counted cones at every visit. If, between years, a tree had died, could not be relocated, or did not bear cones, we randomly chose a replacement tree. As long as there were cones at the first visit, we did not replace a tree that lacked cones on subsequent visits within that year.

*Forest composition and structure*. We used a modified point quarter method, once per location, to determine tree species, density, frequency, basal area, and importance value at survey points [31]. We conducted a point quarter at the survey point, and at four additional locations, 30–35 m to the northwest, northeast, southwest and southeast. At each of the five locations, we divided the area into four quadrants, along north-south and east-west axes. In each quadrant, we documented the species of, and measured the circumference of (± 0.1 cm), and distance to (m) the closest live tree, and the closest live and dead whitebark pine trees. If no trees in a category were present within 200 m, we labeled the quadrant empty. We noted elevation, slope, aspect, and general site conditions at each point.

## Statistical analyses

### Habitat variables

We calculated the number of whitebark pine cones per hectare associated with each nutcracker survey, as the number of cone-bearing whitebark pines per hectare multiplied by the average number of cones. We used the average number of cones at a point counted on the survey date, or, for 2009, the date closest in time to the nutcracker survey.

We used importance value as the measure of local habitat because it incorporates relative frequency, relative density, and relative cover, instead of assuming one of the three variables is an adequate measure. Previous Clark’s nutcracker surveys used basal area as a measure of local whitebark pine habitat [18,19], and as a surrogate for cone crop [18,24,25]. Therefore, we computed Spearman rank correlations between basal area per hectare and importance value, as well as basal area and cone crop (average of first cone count per point per year, across all years).

We determined the area of whitebark pine and Douglas-fir habitat on the landscape with a land cover type map in ArcGIS (10.1, ESRI), using map data from the whitebark pine stand-level condition assessment [24], and four national forest, national park and GAP analysis maps. For details see Schaming [21]. We calculated the area of whitebark pine habitat within a 32.6 km radius buffer around each point. Clark’s nutcrackers travel up to 32.6 km from their summer home range to harvest seeds [17]; although these observations occurred in the Cascades, and nutcrackers are also known to travel shorter distances, this is the maximum distance nutcrackers are known to travel to harvest stands. We also conducted Spearman rank correlations between the area of whitebark pine within 1 km and each other buffer, 2–33 km (2009–2012 sample points; n = 103); all *r*_s_ were >0.70, so we considered the buffer appropriate [32].

We calculated the area of Douglas-fir within 3.2 km of each point, the median diameter of a breeding season home range [23]. The maximum known seed dispersal distance of Douglas-fir is unknown, but we primarily observed nutcrackers foraging on Douglas-fir seeds within the harvest stand during post-harvest and breeding seasons, not conducting long-distance harvest stand to cache site flights with Douglas-fir seeds.

We evaluated if managers could reasonably use importance value, a relatively stable measure of whitebark pine habitat, as a proxy for cone crop. For all points with whitebark pine, we evaluated if the importance value predicted the average number of cones per hectare (using the first count per point per year (n = 5 years)), using a zero-inflated negative binomial model within the “pscl” package [33] in R. Additionally, we fit a negative binomial model to evaluate if cone crop in 2009 predicted cone crop in 2011, using the “MASS” package [34] in R (n = 21, the largest set of paired points at which >0 cones were observed).

We used R to perform all analyses, unless otherwise stated. We checked for normality and homogeneity of variance, and met all key assumptions underlying application of general linear and general linear mixed models. We applied *p* ≤0.05 as the significance level, and report means ± standard error of the mean.

### Occupancy models

We used occupancy models in a Bayesian framework and cross-product model selection (see below and S1 Text) to evaluate whether whitebark pine cone crop, or whitebark pine or Douglas-fir at local or landscape scales influenced Clark’s nutcracker occurrence. Our model integratively analyzes data from all years and each stage of the Clark’s nutcracker annual cycle, allowing for sharing of data across years and stages, while also investigating the importance of covariates hypothesized to be biologically relevant for each stage (Table 2, see S1 Text for full model description) [21,23]. Using detection-nondetection data from repeated site counts, we estimated site- and stage-specific detection (p) and occurrence probability (ψ). Clark’s nutcrackers have large home ranges relative to the sampling location and observer detection range; therefore, nondetection relates to the compound probability of both detection and the probability of being in the part of the home range being sampled. We have no reason to believe that this availability process is non-random, and therefore, relax the interpretation of occupancy to “use” and detection as “available for detection and detected” [30].

**Table 2:** Predictor variables originally included in the single-season occupancy models.

Predictor variables	Breeding season	Early summer	Late summer	Fall harvest	Post-harvest
**Probability of occurrence**					
WBP* cone crop density			x†	x†	
WBP importance value		x	x	x	
Area of WBP on landscape	x†	x†	x†	x†	x†
Douglas-fir importance value	x				
Area of Douglas-fir on landscape	x†				x†
WBP cone crop density X** WBP importance value			x	x	
WBP cone crop density **X** Area of WBP on landscape			x	x	
WBP importance value **X** Area of WBP on landscape		x	x	x	
Douglas-fir importance value **X** Area of WBP on landscape	x				
Area of WBP on landscape **X** Area of Douglas-fir on landscape	x				x
**Detection**					
Tree density	x	x	x	x	x
WBP importance value				x	
Tree density **X** WBP importance value				x	

* WBP is the abbreviation for whitebark pine.

** X signifies an interaction.

^†^ Included in final analysis.

We modeled year- and stage-specific detection probability as a random effect with a single mean and standard deviation, and included an additional observation level random effect to account for lack of model fit [35]. We treated each year as independent because we had predicted *a priori* that regional abundance and behavior of birds that influenced detection may have differed between both years and stages. We modelled year and stage covariate effects as covariate-specific random effects, i.e., with a covariate-specific mean and standard deviation. In addition, when modeling detection, if we visited a point >1 days during a stage in the same year, we only included occurrence data from the first visit (the first set of three ten-minute surveys). We included ‘site’ as a random effect to account for repeated visits to the same site during the same stage over multiple years.

We adopted a Bayesian analysis of the occupancy model to accommodate the year-stage model structure and the use of random effects [36]. We fit a model for each survey radius, ≤100 m and an infinite distance (as far as we could see/hear). We fit the data with JAGS [37] through the “jagsUI” package [38] in R (See S1 Text for a detailed model description and code). We used uninformative priors for all hyperparameters, and posterior distributions were approximated using three 60,000 *Markov chain Monte Carlo (MCMC)* iterations with a burn-in of 20,000 iterations, and thinning rate of 1 (See S1 Text). Chains were visually diagnosed to confirm convergence.

To identify important variables, we used cross-product Bayesian model selection approach [39], a version of variable selection where models, rather than variables, are selected using a latent indicator variable (See S1 Text). Bayesian model selection was not required for detection, because no covariates were included in the *final* detection model (See S1 Text). Based on Barbieri and Berger’s [40] threshold for the mean posterior inclusion probability we excluded all covariates that received an inclusion probability of *P*(*I*_*j*_ = 1 | ***y***) ≤0.5. This criterion is useful, and recommended, for reducing the model space by removing variables with a small marginal inclusion probability [40,41]. For occurrence, at both radii considered, the final global candidate model set included four predictor variables. The model space for each stage was further refined based on the *a priori* predictions, leading to a subset of candidate models used in the final analysis (Table 2).

Because whitebark pine cone crop density and whitebark pine and Douglas-fir importance values were extremely zero-inflated, we included each as a binary (present/absent) and continuous (>0) covariate, which allows the intercept of the occurrence model to vary depending on whether or not the first condition is present. For example, by including the whitebark pine importance value covariate as both binary and continuous, we could ask if the probability of occurrence depended on if whitebark pines were present or not. Then, if occurrence was influenced by whitebark pine presence, did the probability of occurrence change in relation to the amount of whitebark pine? Each covariate form only appeared if the other was in the model. We standardized all predictor variables using z-scores, and, using Spearman rank correlations, determined covariates were not correlated in a way that would influence the resulting inference.

We generated a posterior distribution of the binary indicator variable, representing the number of iterations in which each model was selected. This posterior therefore represents the *model probability*, or the probability that model *m* was the best model from the candidate set *M*. The cross-product approach provides a posterior distribution of the identity of the model accepted in each iteration (i.e. of model support), which in turn identifies which covariate effects are in the model. Using this structure, summarizing the posterior distribution for any parameter conditional on whether it is contained within the supported model conveniently yields model averaged estimates. We also computed covariate importance by summarizing the posterior inclusion probability of each covariate across all models. In the final model set for both radii, each parameter was included in an equal number of models.

To assess model fit, we implemented a Bayesian version of the MacKenzie and Bailey [42] goodness-of-fit test (See S1 Text). For both radii models, the 95% Bayesian Credible intervals (BCI) did include 0, suggesting adequate fit (95% BCI = -1.37–0.46 and -0.59–1.65).

### Comparisons with previous research

To compare our results with predictions of McKinney et al. [18] and Barringer et al.’s [19] models, we determined nutcracker naïve occurrence (presence/absence during the three ten-minute counts). We used mixed models (glmer) in the “lme4” package [43] in R (version 3.1.2) [44] to evaluate fall harvest naïve occurrence as a function of cone crop, with site ID and year as random variables. We separately evaluated occurrence at two radii, ≤100 m and an infinite distance, and ran this pair of models with two different data sets; first, all surveys, then, surveys with whitebark pine cones present (we scaled the cone crop data for the final ≤100 m radius model to enable model convergence).

We then explicitly tested our data within McKinney et al.’s [18] linear regression model, y = -0.449+0.019x, and Barringer et al.’s [19] beta regression model, y = (e^(-1.5165+0.03883* x))/(1+e^(-1.5165+0.03883*x)), both of which examine Clark’s nutcracker occurrence (y) as a function of cone production (x). We only used data we collected within their studies’ parameters: infinite radius surveys, whitebark pine cone crop density >0, cone count and nutcracker survey on same date, and surveys July 15 –September 15. We used the same index of cone production, the squared log of the cone crop density per hectare, and converted occurrence data to the variable the previous studies used, the proportion of survey time with ≥1 detection. Each ten-minute count at a point was labelled absent (0) or present (1); the three counts were then summed (for a total of 0–3), and divided by 3. We used Spearman rank correlations to evaluate if there was a correlation between nutcracker occurrence in our study and predictions from McKinney et al.’s [18], then Barringer et al.’s [19] models. We used one-tailed Wilcoxon signed-rank tests to evaluate if the previous models underpredicted the proportion of time with ≥1 detection.

### Data

All of the original data from which this article is based are deposited at Figshare https://figshare.com/articles/Data_for_paper_Clark_s_nutcracker_occurrence/3494312. Four sets of habitat maps were obtained from third parties and are available upon request. Data from the whitebark pine stand-level condition assessment are available from The Greater Yellowstone Whitebark Pine Subcommittee (http://fedgycc.org/WhitebarkPineOverview.htm). The Bridger-Teton National Forest and Grand Teton National Park maps can be obtained from Nancy Bockino (Nancy_Bockino@nps.gov, Grand Teton National Park). The Shoshone National Forest maps can be obtained from Janice Wilson (janicewilson@fs.fed.us, U.S. Forest Service Rocky Mountain Region Regional Office, Geospatial Services). Wyoming GAP analysis vegetation maps are available online from the U.S. Geological Survey National Gap Analysis Program Land Cover Data Portal (http://gapanalysis.usgs.gov/gaplandcover/).

## Results

### Habitat

Habitat varied considerably between the 238 points (Table 3). Whitebark pine cones were present and visible as early as July 12 and as late as September 29. We counted cones at each point an average of 2 years (± 0.07 SEM; range 1–5). The basal area per hectare and importance value of whitebark pine were highly positively correlated (rho = 0.9, *p* <0.001), but basal area per hectare was only moderately positively correlated with the average cone density per hectare (average of the first cone count per point per year, across all years; rho = 0.6, *p* <0.001).

**Table 3:** Habitat variables at 238 random points.

	Mean ± SEM	Median	Range	# Sites where present versus absent
Whitebark pine cone crop density (cones/ha) [all points; only points with cones]*	258 ± 28; 916 ± 48	0	0–8,132	82 versus 156 (>0 cones in ≥1 year versus 0 cones in all years)
Basal area/ha [all points; only points with whitebark pine]	1.3 ± 0.2; 2.6 ± 0.3	0.03; 1.0	0–18.5; 0.006–18.5	NA
Whitebark pine importance value	57 ± 5	16	0–305	122 versus 115 (importance value >0 versus 0; 1 NA)
Douglas-fir importance value	51 ± 5	0	0–300	81 versus 156 (importance value >0 versus 0; 1 NA)
Area of whitebark pine within 32.6 km (ha)	44,997 ± 1,294	44,068	2,916–87,259	NA
Area of Douglas-fir within 3.2 km (ha)	324 ± 24	228	0–1,754	NA

*Includes cone counts for the first survey per stage of the annual cycle each year, then first survey per stage when cones were present.

The sub-watershed was the minimum mapping unit for the landscape scale measure of whitebark pine, and therefore, the data could not reliably be used for stand-level calculations [45]. However, whitebark pine at the local scale measured in the field, and landscape scale determined via ArcGIS, were reasonably consistent. The mean distance to whitebark pine habitat for points with whitebark pine present (i.e. importance value >0) was 0.1 ± 0.02 km (n = 247; range = 0–1.5), but was 2.0 ± 0.1 km (n = 201; range = 0–7.3) if no whitebark pine was present (i.e. importance value = 0).

### Predictability of whitebark pine cone crop

At sites with whitebark pine, higher whitebark pine importance value significantly predicted a higher average number of whitebark pine cones (n = 122, β = 0.009 ± 0.003, p = 0.0004); the log odds of number of cones would increase an estimated 0.003–0.015 per unit increase in importance value. The three outliers did not alter significance. However, the number of cones at a point in 2009 did not predict the number of cones at the same point in 2011 (n = 21; β = 0.00005 ± 0.0002, p = 0.8).

### Occupancy models

**Fig 2:**
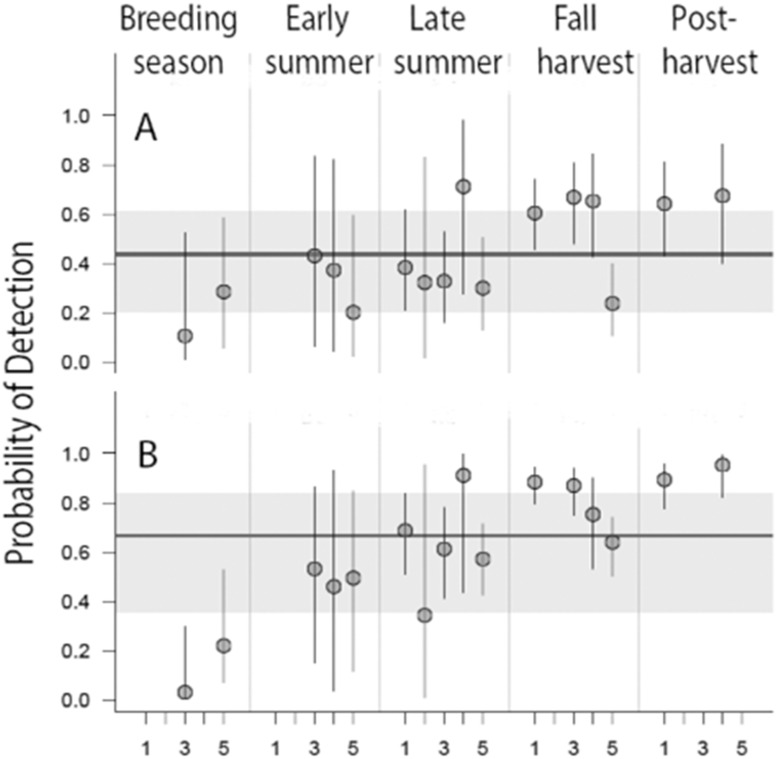
Posterior mean year-stage detection probabilities (red circles) and 95% Bayesian Credible Intervals (BCI; vertical black lines) of the (A) ≤100 m radius, and (B) infinite radius data. Year-stage parameters are random effects from a hyper-distribution represented by the blue horizontal line (the posterior mean of the mean), the blue shaded area represents the 95% BCI of the estimate mean. Numbers 1–5 represent 2009–2013.

Occupancy models included data from 2,526 surveys (Table 1). Detection varied across stages and years with a general trend of increasing detectability through the year. Detection was relatively low during the breeding season, moderate during the early and late summer, and relatively high during the fall harvest and post-harvest stages (Table 4 and Fig 2). Within-year variation was highest in late summer and certainty in detection probabilities reflected variation in sample sizes (Table 4 and Fig 2).

**Table 4:** Posterior mean year-stage detection probabilities and 95% Bayesian Credible Intervals. Dashes indicate stage-year combinations without data.

Year	2009	2010	2011	2012	2013
Fall whitebark pine cone crop [46]	very high	low	medium	high	low
**≤100 m radius**					
Breeding season	-	-	0.10 (0.01,0.52)	-	0.27 (0.06,0.58)
Early summer	-	-	0.41 (0.07,0.83)	0.36 (0.05,0.82)	0.19 (0.02,0.6)
Late summer	0.39 (0.21,0.62)	0.30 (0.02,0.83)	0.32 (0.16,0.53)	0.74 (0.28,0.98)	0.29 (0.13,0.5)
Fall harvest	0.60 (0.46,0.74)	-	0.67 (0.48,0.81)	0.66 (0.43,0.84)	0.23 (0.11,0.4)
Post-harvest	0.64 (0.43,0.81)	-	-	0.68 (0.4,0.88)	-
**Infinite radius**					
Breeding season	-	-	0.03 (0,0.3)	-	0.22 (0.07,0.53)
Early summer	-	-	0.53 (0.15,0.87)	0.45 (0.04,0.93)	0.49 (0.12,0.85)
Late summer	0.69 (0.51,0.84)	0.33 (0.01,0.95)	0.61 (0.41,0.78)	0.92 (0.44,1)	0.57 (0.43,0.71)
Fall harvest	0.88 (0.79,0.94)	-	0.87 (0.75,0.94)	0.75 (0.53,0.9)	0.64 (0.5,0.74)
Post-harvest	0.89 (0.78,0.96)	-	-	0.95 (0.82,0.99)	-

Clark’s nutcracker occurrence varied as a function of habitat variables differently depending on stage of the annual cycle (Tables 5 and 6). The presence/absence of whitebark pine cones, and area of whitebark pine and Douglas-fir on the landscape affected occurrence during some, but not all stages. However, model averaged, or conditional, parameter estimates suggested the density of whitebark pine cones, whitebark pine importance value, and Douglas-fir importance value were not important in predicting occurrence at any stage, as compared to other variables in the model sets.

**Table 5:** Stage-specific posterior occupancy model probabilities for final candidate models. M1 –M6 are models 1–6. Dashes indicate stages in which effects were constrained to be 0 (i.e. were not considered).

	M1	M2	M3	M4	M5	M6
**Covariates**						
Intercept	X	X	X	X	X	X
Whitebark pine cone crop density (binary)		X			X	
Whitebark pine cone crop density (continuous)		X			X	
Area of whitebark pine on the landscape			X		X	X
Area of Douglas-fir on the landscape				X		X
**Stage of the annual cycle**						
**≤100 m radius**						
Breeding season	0.12	-	0.03	0.74	-	0.18
Early summer	0.70	-	0.30	-	-	-
Late summer	0.09	0.50	0.27	-	0.13	-
Fall harvest	0.00	0.29	0.08	-	0.63	-
Post-harvest	0.50	-	0.15	0.24	-	0.11
**Infinite radius**						
Breeding season	0.02	-	0.00	0.69	-	0.29
Early summer	0.81	-	0.19	-	-	-
Late summer	0.23	0.34	0.30	-	0.14	-
Fall harvest	0.03	0.50	0.11	-	0.36	-
Post-harvest	0.28	-	0.10	0.34	-	0.29

**Table 6:** Model averaged, or conditional, parameter estimates (± 95% credible intervals), and conditional posterior support for each parameter in the occupancy model. Posterior distribution of stage-specific parameter estimates, and conditional posterior support which shows how important is each covariate in predicting occurrence as compared to other parameters in the model set (in square brackets). Dashes indicate stages in which effects were constrained to be 0 (i.e. were not considered). Bolded results indicate that the CI’s exclude 0 (i.e. there is an effect).

	Intercept	Whitebark pine cone crop density (binary)*	Whitebark pine cone crop density (continuous)	Area of whitebark pine on the landscape	Area of Douglas-fir on the landscape
**≤100 m radius**					
Breeding season (Fig 3)	-0.86 (-1.93,1.42) [1.00]	-	-	0.39 (-0.58,2.23) [0.21]	**-1.39 (-3.09,-0.32) [0.92]**
Early summer	-1.03 (-2.46,1.26) [1.00]	-	-	-0.83 (-2.63,0.37) [0.30]	-
Late summer (Fig 4)	2.72 (0.62,6.09) [1.00]	**-2.51 (-5.46,-0.32) [0.63]**	-0.37 (-2.07,1.52) [0.63]	1.25 (-0.05,3.72) [0.40]	-
Fall harvest (Fig 5)	4.23 (2.37,6.84) [1.00]	**-3.61 (-6.28,-1.37) [0.92]**	0.57 (-1.82,2.4) [0.92]	**0.83 (0.15,2.53) [0.71]**	-
Post-harvest (Fig 6)	2.09 (0.69,5.79) [1.00]	-	-	0.9 (-1.70,4.63) [0.26]	0.78 (-3.41,4.18) [0.35]
**Infinite radius**					
Breeding season	0.27 (-1.14,2.79) [1.00]	-	-	0.87 (-0.24,2.54) [0.29]	**-2.06 (-4.77,-0.63) [0.98]**
Early summer	-0.5 (-1.38,0.7) [1.00]	-	-	-0.46 (-1.49,0.37) [0.19]	-
ate summer	3.75 (1.44,7.14) [1.00]	-2.36 (-5.2,0.99) [0.48]	-0.27 (-2.45,1.86) [0.48]	1.25 (-2.33,3.72) [0.44]	-
Fall harvest	5.22 (3.07,8.06) [1.00]	**-3.44 (-6.26,-1.06) [0.86]**	-0.08 (-2.69,1.94) [0.86]	**0.83 (0.01,2.25) [0.47]**	-
Post-harvest	2.87 (1.36,5.31) [1.00]	-	-	1.11 (-0.69,3.32) [0.39]	2.36 (-0.89,6.23) [0.63]

* Cones present = 0, cones absent = 1.

**Fig 3:**
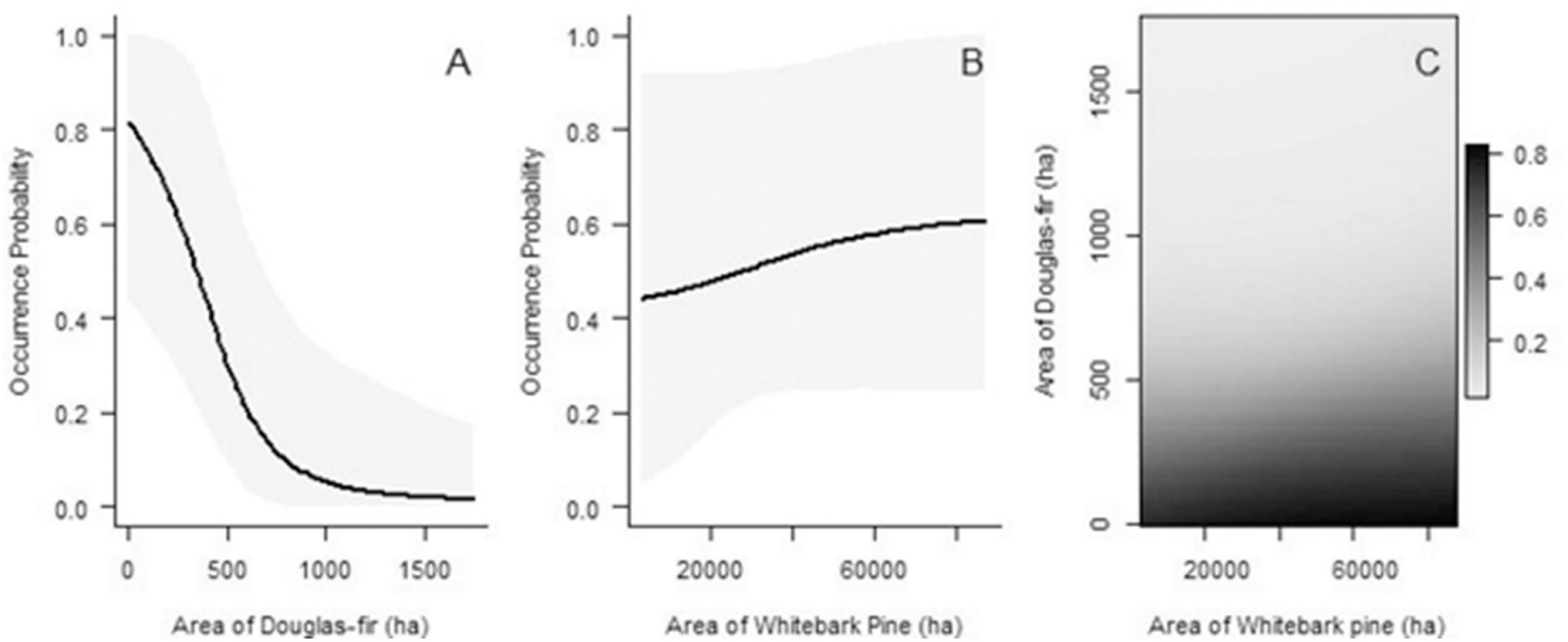
Predicted probability of Clark’s nutcracker occurrence during the breeding season as a function of area of (A) Douglas-fir, (B) whitebark pine, and (C) both species on the landscape. The graphs depict predictions based on data from infinite radius surveys. Values represent area of whitebark pine habitat within 32.6 km and Douglas-fir habitat within 3.2 km. On each graph, all variables in the models that are not shown on the graphs, are held at the mean. Gray shaded areas denote 95% Bayesian Credible Intervals.

#### Seasonal occurrence

**Fig 4:**
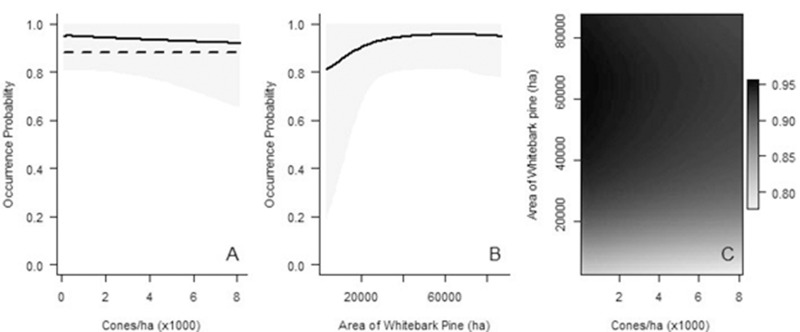
Predicted probability of Clark’s nutcracker occurrence during the late summer as a function of the (A) whitebark pine cones/ha, (B) area of whitebark pine on the landscape, and (C) both metrics. The graphs depict predictions based on data from infinite radius surveys. Values represent area of whitebark pine habitat within 32.6 km. On each graph, all variables in the models that are not shown on the graphs, are held at the mean. Gray shaded areas denote 95% Bayesian Credible Intervals. In A, the reference horizontal line shows the intercept when no cones are present.

*Late summer*. Presence/absence of cones at ≤100 m, but not at an infinite radius, had a relatively strong influence on nutcracker occurrence (Tables 5 and 6). Depending on the radius, the presence or absence of cones was 7–9 times more important than density of whitebark pine cones (Table 6). At ≤100 m there was a 27–99.6% increase in the odds of a site being occupied if cones were present, regardless of the density. However, at an infinite radius, the results were equivocal as the confidence intervals bounded zero. At all cone crop levels, when a mean area of whitebark pine is present on the landscape, the probability of occurrence is predicted to be high, although there is relatively high uncertainty at higher cone crops (Fig 4). A high probability of occurrence, ≥75%, is predicted when the landscape is composed of a minimum of 8% cone-bearing whitebark pine habitat (~25,000 ha) within a 32.6 km radius.

*Fall harvest*. At ≤100 m and infinite radii, presence/absence of cones, and area of whitebark pine on the landscape both had a strong influence on Clark’s nutcracker occurrence. Whether or not cones were present was 6–43 times more important than the number of whitebark pine cones (Table 6). There was a 75–99.8% and 65–99.8% increase in the odds of a site being occupied if cones were present, respectively.

**Fig 5:**
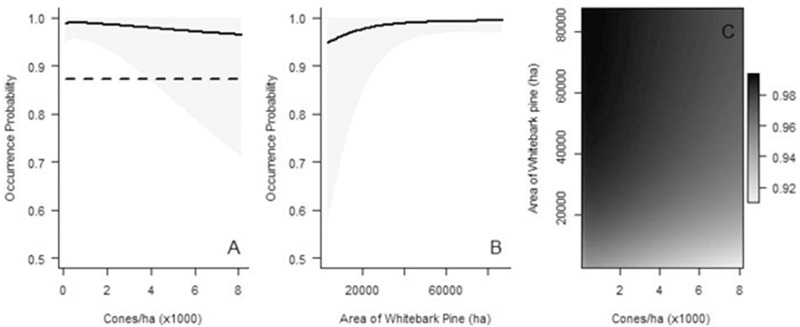
Predicted probability of Clark’s nutcracker occurrence during the fall harvest as a function of the (A) whitebark pine cones/ha, (B) area of whitebark pine on the landscape, and (C) both metrics. The graphs depict predictions based on data from infinite radius surveys. Values represent area of whitebark pine habitat within 32.6 km. On each graph, all variables in the models that are not shown on the graphs, are held at the mean. Gray shaded areas denote 95% Bayesian Credible Intervals. In A, the reference horizontal line shows the intercept when no cones are present.

As cone crop increases, when a mean area of whitebark pine is present on the landscape, the probability of occurrence is predicted to be high and change very little (Fig 5). However, when the minimum area of whitebark pine is present, Bayesian credible intervals for the probability of occurrence are high. Accounting for variability, a ≥75% probability of occurrence is predicted when the landscape is composed of a minimum of 4% cone-bearing whitebark pine habitat (~12,000 ha) within a 32.6 km radius. At a high level of cone crop, there is a high uncertainty of probability of occurrence regardless of the area of whitebark pine.

**Fig 6:**
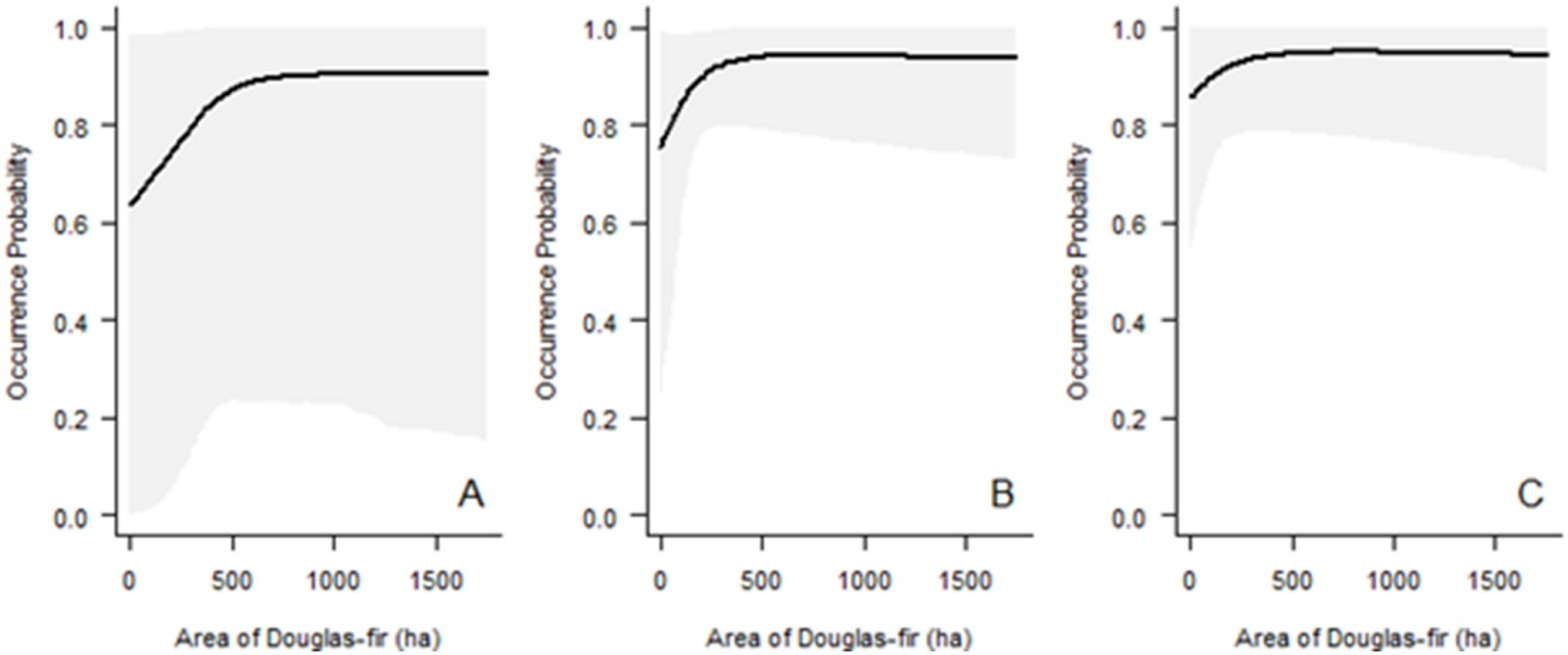
Predicted probability of Clark’s nutcracker occurrence during the post-harvest as a function of the area of Douglas-fir and whitebark pine on the landscape. The graphs depict predictions based on data from infinite radius surveys. Values represent area of Douglas-fir habitat within 3.2 km. Area of whitebark pine is held at the (A) minimum, (B) mean, and (C) maximum, and all other variables are held at the mean. Gray shaded areas denote 95% Bayesian Credible Intervals.

*Post-harvest*. Clark’s nutcracker occurrence is predicted to increase, then plateau, with an increase in the area of Douglas-fir on the landscape (Fig 6). Bayesian credible intervals are high when there is a relatively small area of whitebark pine, and the highest when there is low area of both Douglas-fir and whitebark pine.

### Comparison with previous research

**Fig 7:**
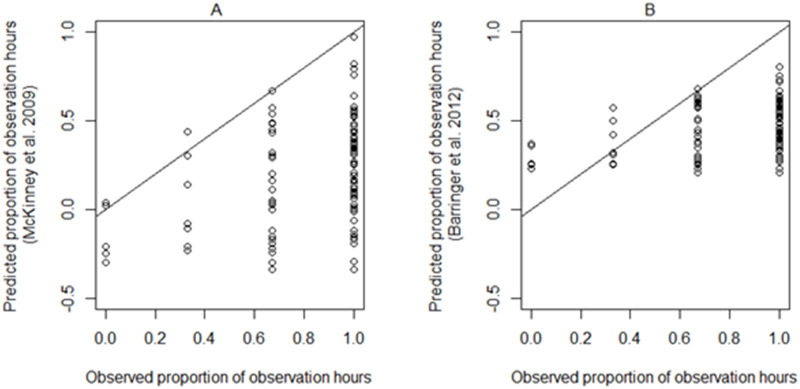
Observed Clark’s nutcracker occurrence versus predictions using (A) McKinney et al.’s [18] and (B) Barringer et al.’s [19] models. The diagonal line represents a perfect prediction from the model (1:1). Previous studies consistently underpredicted the proportion of time Clark’s nutcrackers were detected.

Fall harvest naïve occurrence was 45% for ≤100 m radius and 71% for infinite radius surveys (n = 450). Occurrence significantly increased with whitebark pine cone density (≤100 m radius: β = 0.002 ± 0.0006, *p* = 0.008; infinite radius: β = 0.004 ± 0.002, *p* = 0.03). However, when only comparing sites with whitebark pine cones (n = 118), the cone density did not significantly predict occurrence (≤100 m radius: β = 0.6 ± 0.5, p = 0.3; infinite radius: β = 0.002 ± 0.002, p = 0.3). Fall harvest nutcracker occurrence (infinite radius) was only weakly positively correlated with that predicted by McKinney et al. [18] and Barringer et al. [19] (n = 110; rho = 0.3, p = 0.008, and rho = 0.3, p = 0.006). Both previous models significantly underpredicted nutcracker occurrence in our study (n = 110; W = 714.5, *p* <0.0001, and W = 1096.5, *p* <0.0001, respectively; Fig 7).

## Discussion

### Management implications

#### Influence of whitebark pine metrics on occurrence

Under current conditions in the southern Greater Yellowstone Ecosystem, Clark’s nutcracker occurrence during the fall seed harvest season increases with both the presence of cone-bearing whitebark pine trees, and a higher area of whitebark pine habitat on the landscape. Clark’s nutcracker occurrence in our study is far more strongly influenced by presence of whitebark pine cones rather than cone density. In contrast, previous research has indicated that occurrence was associated with whitebark pine cone density and basal area [18,19]. As a consequence, whitebark pine management efforts have focused on reaching a cone density of at least ~1,000 cones per ha or a basal area of 2–5 m^2^ per ha, the densities predicted to yield a high probability of nutcracker occurrence [18,19,24,25].

Instead, to encourage a high, >75%, probability of Clark’s nutcracker occurrence in this region, we suggest that management would be served by ensuring the landscape is composed of a minimum of 4–8% cone-bearing whitebark pine habitat (~12,500–25,000 ha) within a 32.6 km radius of a priority area, such as a stand where whitebark pine management actions are proposed. Additionally, rather than attempting to achieve a specific cone and basal area density within a stand, managers could simply focus on increasing whitebark pine cone densities and basal area in stands. During the fall harvest, Clark’s nutcrackers continue harvesting and caching whitebark pine seeds as long as they are available, and during large cone crops, individuals have been estimated to cache 3–5 times more seeds than they need for their energetic requirements [16,47]. A lower cone crop leads to a shorter harvest, which likely translates into a lower number of seeds dispersed and a higher proportion of seeds retrieved per bird. Accordingly, at the current Clark’s nutcracker population level, at higher cone densities in some stands, the higher variability in nutcracker occurrence should result in a longer harvest. Higher numbers of cached seeds and fewer retrieved caches would increase the potential for whitebark pine regeneration.

#### Influence of Douglas-fir metrics on occurrence

We suggest that management focus on restoring whitebark pine in a mosaic with Douglas-fir in the Greater Yellowstone Ecosystem, and, by extrapolation, alternate nutcracker seed sources, such as limber pine, Jeffrey pine (*Pinus jeffreyi*), or ponderosa pine (*Pinus ponderosa*), in other regions [48]. During the post-harvest stage, higher nutcracker occurrence was associated with a moderate to high area of whitebark pine and a moderate area of Douglas-fir. In fact, low occurrence was only likely at sites with both low whitebark pine and low Douglas-fir habitat. These results suggest that nutcrackers were not moving out of the whitebark pine habitats once the cone crop was depleted, and Douglas-fir habitat, which provided an alternate seed source, was primarily used when in a habitat mosaic with whitebark pine.

On the other hand, during the breeding season, Clark’s nutcracker occurrence decreased with a higher area of Douglas-fir, regardless of the whitebark pine area. This was surprising because radio-tagged Clark’s nutcrackers in the study area highly selected Douglas-fir habitat for their breeding season home ranges [23]. However, although radio-tagged Clark’s nutcrackers selected Douglas-fir habitat from the available habitat on the landscape, it only made up an average of 22% of the total habitat within each bird’s home range (n = 55) [21], and when selecting habitats within the home ranges, the birds used Douglas-fir in proportion to availability [23]. The occurrence patterns reveal which resources are important drivers of nutcracker distribution and population dynamics, whereas habitat selection is the process by which individual birds select habitats relative to their availability [30,49]. The seemingly contradictory habitat use and selection results suggest that a habitat mosaic which contains a small proportion of Douglas-fir is optimal. We suggest targeting whitebark pine management within stands adjacent to or within 3.2 km of Douglas-fir stands (3.2 km is the median diameter of a nutcracker breeding season home range [23]).

#### Comparisons with previous models

Both McKinney et al. [18] and Barringer et al.’s [19] models underpredicted nutcracker detections in our study. In all three studies, higher naïve Clark’s nutcracker observations during the fall harvest were associated with higher whitebark pine cone density. However, once we used occupancy models to account for detectability, and included both presence/absence of cones and area of whitebark pine, in addition to cone crop density, nutcracker occurrence was only weakly associated with the density of whitebark pine cones. The variation in study design may also partially account for discrepancies in predicted results (S2 Text). Additionally, in our study we evaluated data separately for five stages, each based on Clark’s nutcracker behavior associated with their annual cycle and annual environmental changes. The previous studies combined late summer and fall survey data, but we discovered the influence of whitebark pine metrics on occurrence varied considerably during these two periods. We also included both presence/absence of cones and cone crop density in our models because, like much ecological data, our cone densities were zero-inflated, and therefore sites without cones were likely to have a large impact. By doing this, we were able to conclude how much more important simple presence of cones was on Clark’s nutcracker occurrence. By using model selection to compare models which included one or more habitat variables, we determined that models which included both cone crop and area of whitebark pine on the landscape during the fall harvest were better supported than models with only cone crop. This suggests that area of whitebark pine on the landscape should be taken into account when setting management goals. We suggest nutcrackers would be less likely to occupy isolated stands, even if they have a live whitebark pine basal area of 2–5 m^2^/ha [18,19], if there is not an adequate amount of whitebark pine on the landscape. Due to the contradictions between our results and those of previous research, we suggest that management should reconsider basing their whitebark pine restoration strategies on the earlier models, at least in the southern Greater Yellowstone Ecosystem. We are not suggesting abandoning current whitebark pine management strategies throughout the Clark’s nutcracker- whitebark pine range, but instead urge caution in using the specific basal area and whitebark pine cone thresholds suggested by previous models. Specific, easy-to-implement solutions may be ideal, but are not necessarily possible. Retaining Clark’s nutcrackers as natural seed dispersers is important, and it is reasonable to assume that increasing area of whitebark pine habitat, increasing basal area of whitebark pines trees, and increasing number of cones, will, overall, positively impact nutcracker occurrence. We suggest Clark’s nutcracker habitat use should be separately evaluated in each ecosystem of conservation interest, or at a minimum in a greater number of ecosystems. Additionally, although most management actions occur at a stand scale, what a “stand” constitutes is not necessarily clear, and there should be greater focus on evaluating the appropriate spatial scale at which to manage whitebark pine.

#### Influence of detectability

Our results emphasize the importance of accounting for detectability of a species when predicting probability of occurrence. In our study, failing to consider detectability would have underestimated true occupancy rates [29]. By extrapolation, we suggest that the data used to generate the previous models underestimated true occupancy rates. When considering conservation strategies, it is not necessarily detrimental to underestimate occupancy, but it could lead to misallocated resources, or a misunderstanding of the system.

Clark’s nutcracker detectability varied between years and stages of the annual cycle, but due to the small sample size of 2–5 years for each stage, we were unable to examine which annual variables influenced detection rates. Cone crop may have had an indirect effect on Clark’s nutcracker occurrence as Clark’s nutcrackers are known to move out of an ecosystem in years with widespread low to moderate cone crops [50,51], and a lower number of birds may have decreased detectability. Also, population-wide nonbreeding occurs in the population [21]. We observed that breeding birds were more secretive and quiet, and less likely to travel in flocks. Therefore, in years when birds did not breed, detectability may have been higher. The variation in detectability between stages was likely a result of consistent changes in Clark’s nutcracker foraging and social behavior throughout the year [48]; however, using the random effects structure may have captured this between- and within-year variability.

### Stability of the Clark’s nutcracker-whitebark pine mutualism

In the Greater Yellowstone Ecosystem, the mountain pine beetle outbreak between the early 2,000s and ~2010 killed approximately 75% of the mature whitebark pine trees [20,52]. Trees of all sizes continue to be infected and killed by white pine blister rust, which is now persistent and pervasive in the region [53]. The interaction of bark beetles, white pine blister rust, changing fire regimes, climate change, competitors, and Clark’s nutcracker behavior make whitebark pine particularly vulnerable to decline, particularly in the face of climate change [54]. However, even with drastic recent declines of whitebark pine, a high probability of Clark’s nutcracker presence was associated with presence of cone bearing whitebark pine trees during the fall harvest season, suggesting the birds are available to disperse whitebark pine seeds, even at low cone crop densities. Similarly, previous results from the Greater Yellowstone, the Cascade Range and Glacier National Park, a location with a relatively low population of Clark’s nutcrackers, suggested Clark’s nutcrackers foraged in whitebark pine stands with low cone densities [19,55,56]. However, it is unclear if this high occurrence is associated with high levels of whitebark pine seed dispersal.

Clark’s nutcrackers are facultative migrants, and have evolved to regularly move over the larger landscape, tracking food resources [51]. By observing Clark’s nutcrackers during surveys throughout the year, we suggest that there is an adequate amount of seeds available for caching to maintain an overwintering Clark’s nutcracker population. Additionally, recent 2018 surveys within the Greater Yellowstone Ecosystem show continued whitebark pine recruitment, with an average of 55 small whitebark pines per each 500 m^2^ transect [57]. As virtually all germinating whitebark pine seedlings sprout from nutcracker caches, this recruitment shows that the mutualism, at least in the recent past, continues to function. We suggest additional studies of whitebark pine seedling density to help evaluate continued function of the Clark’s nutcracker-whitebark pine mutualism within the region.

### Limitations and opportunities

The complication with suggesting management recommendations based on results from this study and the few recent studies of nutcracker occurrence patterns, is that each study only describes the relationship between Clark’s nutcrackers and habitat at a snapshot in time [18,19,55,56]. Historical habitat use is unknown. Therefore, we do not know if what we observed is representative of the past, before large-scale declines of whitebark pine and other conifer habitats, or at varying Clark’s nutcracker population sizes. Population size, individual fitness and behaviors, including habitat use and selection, can all vary with density of the species and habitats involved in interactions [58,59]. If the local Clark’s nutcracker or whitebark pine populations are higher or lower, how does the relationship change?

Predictions of thresholds necessary for continued Clark’s nutcracker-whitebark pine stability are based on current conditions, and may be overly simplistic. To improve long-term management outcomes, we therefore suggest adopting an adaptive management approach [60,61]. We suggest continued monitoring of the relationship between Clark’s nutcrackers and whitebark pine as environmental conditions change and management strategies are implemented, so that the predictions can be modified and revised with the new information. Because of the conflicting results between Clark’s nutcracker habitat use and habitat selection, we recommend a greater focus on differentiating preference versus prevalence. Due to the high mobility of Clark’s nutcrackers and the large-scale declines of many of their habitats, we also suggest monitoring Clark’s nutcracker habitat associations range-wide. An increased focus on the status of the Clark’s nutcracker metapopulation will allow more robust predictions of stability of the Clark’s nutcracker-whitebark pine mutualism.

## Supporting information

S1 Text Appendix 1. (PDF)

S2 Text Appendix 2. (PDF)

## References

[1] Case BS, Buckley HL, Barker-Plotkin AA, Orwig DA, Ellison AM. When a foundation crumbles: forecasting forest dynamics following the decline of the foundation species Tsuga canadensis. Ecosphere. 2017;8: e01893.

[2] Dominguez-Begines J, De Deyn GB, Garcia LV, Eisenhauer N, Gomez-Aparicio L, Allan E. Cascading spatial and trophic impacts of oak decline on the soil food web. J Ecol. 2019; 1199.

[3] Manenti R, Ghia D, Fea G, Ficetola GF, Padoa-Schioppa E, Canedoli C. Causes and consequences of crayfish extinction: Stream connectivity, habitat changes, alien species and ecosystem services. Freshw Biol. 2019;64: 284–293.

[4] Tomback DF, Arno SF, Keane RE. Whitebark pine communities: ecology and restoration. Washington D.C. USA: Island Press 2001.

[5] Tomback DF, Resler LM, Keane RE, Pansing ER, Andrade AJ, Wagner AC. Community Structure, Biodiversity, and Ecosystem Services in Treeline Whitebark Pine Communities: Potential Impacts from a Non-Native Pathogen. Forests. 2016;7: 21.

[6] Ellison AM, Bank MS, Clinton BD, Colburn EA, Elliott K, Ford CR, et al Loss of foundation species: consequences for the structure and dynamics of forested ecosystems. Front Ecol Environ. 2005;3: 479–486.

[7] Mattson DJ, Blanchard BM, Knight RR. Yellowstone grizzly bear mortality, human habituation, and whitebark pine seed crops. J Wildl Manag. 1992; 432–442.

[8] Tomback DF, Achuff P. Blister rust and western forest biodiversity: ecology, values and outlook for white pines: Blister rust and western forest biodiversity. For Pathol. 2010;40: 186–225.

[9] Gibson K. Mountain Pine Beetle Conditions in Whitebark Pine Stands in the Greater Yellowstone Ecosystem, USDA Forest Service, Forest Health Protection. Rpt. 06–03. Missoula Field Office, Missoula, Montana. 2006.

[10] Department of the Interior. Federal Register. Endangered and Threatened Wildlife and Plants; Review of Native Species That Are Candidates for Listing as Endangered or Threatened; Annual Notice of Findings on Resubmitted Petitions; Annual Description of Progress on Listing Actions Vol. 80. No. 247. 2015. Available: https://www.gpo.gov/fdsys/pkg/FR-2015-12-24/pdf/2015-32284.pdf.

[11] Government of Canada. Whitebark Pine, Species Profile, Species at Risk Public Registry. 2016. https://wildlife-species.canada.ca/species-risk-registry/species/speciesDetails_e.cfm?sid=1086.

[12] Resler LM, Tomback DF. Blister Rust Prevalence in Krummholz Whitebark Pine: Implications for Treeline Dynamics, Northern Rocky Mountains, Montana, U.S.A. Arct Antarct Alp Res. 2008;40: 161–170.

[13] Hutchins HE, Lanner RM. The central role of Clark’s nutcracker in the dispersal and establishment of whitebark pine. Oecologia. 1982;55: 192–201. 28311233 10.1007/BF00384487

[14] Tomback DF. Foraging strategies of Clark’s Nutcracker. Living Bird. 1978;16: 123–160.

[15] Lorenz TJ, Sullivan KA. Seasonal Differences in Space Use by Clark’s Nutcrackers in the Cascade Range. The Condor. 2009;111: 326–340.

[16] Tomback DF. Dispersal of whitebark pine seeds by Clark’s nutcracker: a mutualism hypothesis. J Anim Ecol. 1982;51: 451–467.

[17] Lorenz TJ, Sullivan KA, Bakian AV, Aubry CA. Cache-site selection in Clark’s Nutcracker (Nucifraga columbiana). The Auk. 2011;128: 237–247.

[18] McKinney ST, Fiedler CE, Tomback DF. Invasive pathogen threatens bird-pine mutualism: implications for sustaining a high-elevation ecosystem. Ecol Appl. 2009;19: 597–607. 19425424 10.1890/08-0151.1

[19] Barringer LE, Tomback DF, Wunder MB, McKinney ST. Whitebark Pine Stand Condition, Tree Abundance, and Cone Production as Predictors of Visitation by Clark’s Nutcracker. Newsom LA, editor. PLoS ONE. 2012;7: e37663. 22662186 10.1371/journal.pone.0037663 PMC3360761

[20] Macfarlane WW, Logan JA, Kern WR. An innovative aerial assessment of Greater Yellowstone Ecosystem mountain pine beetle-caused whitebark pine mortality. Ecol Appl. 2013;23: 421–437. 23634592 10.1890/11-1982.1

[21] Schaming TD. Population-Wide Failure to Breed in the Clark’s Nutcracker (Nucifraga columbiana). PLoS ONE. 2015;10: e0123917. 25970294 10.1371/journal.pone.0123917 PMC4430254

[22] Giuntoli M, Mewaldt LR. Stomach contents of Clark’s nutcrackers collected in western Montana. The Auk. 1978;95: 595–598.

[23] Schaming TD. Clark’s Nutcracker Breeding Season Space Use and Foraging Behavior. PLoS ONE. 2016;11: e0149116. 26881774 10.1371/journal.pone.0149116 PMC4755556

[24] Greater Yellowstone Coordinating Committee Whitebark Pine Subcommittee. Whitebark Pine Strategy for the Greater Yellowstone Area. 2011. 41 p. http://fedgycc.org/documents/WBPStrategyFINAL5.31.11.pdf.

[25] Keane RE, Tomback DF, Aubry CA, Bower AD, Campbell EM, Cripps CL, et al A range-wide restoration strategy for whitebark pine (Pinus albicaulis). Gen. Tech. Rep. RMRS-GTR-279. Fort Collins, CO: U.S. Department of Agriculture, Forest Service, Rocky Mountain Research Station 108 p. 2012.

[26] Leirfallom SB, Keane RE, Tomback DF, Dobrowski SZ. The effects of seed source health on whitebark pine (Pinus albicaulis) regeneration density after wildfire. Can J For Res 45 1597–1606. 2015;45: 1597–1606.

[27] Perkins DL, Cochrane AC, Means RE, United States. Conservation and management of whitebark pine ecosystems on Bureau of Land Management Lands in the western United States. [Denver, Colo: U.S. Department of the Interior, Bureau of Land Management]. 2016.

[28] Schaming TD. A keystone mutualism in an altered ecosystem. Dissertation in partial fulfillment of the Ph.D. degree at Cornell University. 2016.

[29] MacKenzie DI, Nichols JD, Lachman GB, Droege S, Andrew Royle J, Langtimm CA. Estimating site occupancy rates when detection probabilities are less than one. Ecology. 2002;83: 2248–2255.

[30] MacKenzie DI, Nichols JD, Royle JA, Pollock KH, Bailey L, Hines JE. Occupancy Estimation and Modeling: Inferring Patterns and Dynamics of Species Occurrence. Academic Press 2006.

[31] Cottam G, Curtis JT. The Use of Distance Measures in Phytosociological Sampling. Ecology. 1956;37: 451–460.

[32] Martin A, Fahrig L. Measuring and selecting scales of effect for landscape predictors in species-habitat models. Ecol Appl. 2012;22: 2277–2292. 23387125 10.1890/11-2224.1

[33] Jackman S. pscl: Classes and Methods for R Developed in the Political Science Computational Laboratory, Stanford University. Department of Political Science, Stanford University. Stanford, California. R package version 1.4.9. URL http://pscl.stanford.edu/. 2015.

[34] Venables WN, Ripley BD. Modern Applied Statistics with S. Fourth Edition Springer, New York 2002.

[35] Harrison X. Using observation-level random effects to model overdispersion in count data in ecology and evolution. PeerJ. 2014;2: e616. 25320683 10.7717/peerj.616 PMC4194460

[36] Kéry M, Royle JA. Inference about species richness and community structure using species-specific occupancy models in the national Swiss breeding bird survey MHB Modeling demographic processes in marked populations. Springer; 2009 pp. 639–656. http://link.springer.com/chapter/10.1007/978-0-387-78151-8_28.

[37] Plummer M. JAGS: A Program for Analysis of Bayesian Graphical Models Using Gibbs Sampling, Proceedings of the 3rd International Workshop on Distributed Statistical Computing (DSC 2003), March 20–22, Vienna, Austria. ISSN 1609-395X. 2003.

[38] Kellner K. Package ‘jagsUI’. A Wrapper Around “rjags” to Streamline “JAGS” Analyses. Version 1.4.2. https://cran.r-project.org/web/packages/jagsUI/jagsUI.pdf. 2016.

[39] Tenan S, O’Hara RB, Hendriks I, Tavecchia G. Bayesian model selection: the steepest mountain to climb. Ecol Model. 2014;283: 62–69.

[40] Barbieri MM, Berger JO. Optimal predictive model selection. Ann Stat. 2004; 870–897.

[41] Ntzoufras I. Bayesian Modeling Using WinBUGS. Hoboken, USA: John Wiley and Sons; 2009.

[42] MacKenzie DI, Bailey LL. Assessing the fit of site-occupancy models. J Agric Biol Environ Stat. 2004;9: 300–318.

[43] Bates D, Maechler M, Bolker B, Walker S. Fitting Linear Mixed-Effects Models Using lme4. Journal of Statistical Software. 2015; 67(1), 1–48.

[44] R Core Team. R: A language and environment for statistical computing. R Foundation for Statistical Computing, Vienna, Austria http://www.R-project.org/. 2013.

[45] Macfarlane WW, Logan JA, Kern WR. Using the Landscape Assessment System (LAS) to Assess Mountain Pine Beetle-Caused Mortality of Whitebark Pine, Greater Yellowstone Ecosystem, 2009: Project Report. Prepared for the Greater Yellowstone Coordinating Committee, Whitebark Pine Subcommittee, Jackson, Wyoming. 2010. http://docs.nrdc.org/land/files/lan_10072101a.pdf.

[46] Haroldson MA (Editor). Whitebark Pine Cone Production, 2015 Project Summary. http://www.nrmsc.usgs.gov/files/norock/products/IGBST/2015WBPSummary.pdf. Interagency Grizzly Bear Study Team; 2015.

[47] Vander Wall SB, Balda RP. Coadaptations of the Clark’s nutcracker and the pinon pine for efficient seed harvest and dispersal. Ecol Monogr. 1977;47: 89–111.

[48] Tomback DF. Clark’s Nutcracker (Nucifraga columbiana) In: Poole A, editor. The Birds of North America Online. Ithaca, New York: Cornell Lab of Ornithology 1998 Available: http://bna.birds.cornell.edu/bna/species/331.

[49] Manly BFJ, McDonald LL, Thomas DL, McDonald TL, Erikson WP. Resource selection by animals: statistical design and analysis for field studies. Dordrecht, The Netherlands: Kluwer Academic Publishers 2002.

[50] Davis J, Williams L. Irruptions of the Clark nutcracker in California. The Condor. 1957;59: 297–307.

[51] Vander Wall SB, Hoffman SW, Potts WK. Emigration behavior of Clark’s Nutcracker. The Condor. 1981;83: 162–170.

[52] Logan J, Macfarlane W, Willcox L. Effective monitoring as a basis for adaptive management: A case history of mountain pine beetle in Greater Yellowstone Ecosystem whitebark pine. IForest Biogeosciences For. 2009;2.

[53] Shanahan E, Irvine KM, Thoma DP, Wilmoth SK, Ray A, Legg K, et al Whitebark pine mortality related to white pine blister rust, mountain pine beetle outbreak, and water availability. Ecosphere. 2016;7.

[54] Hansen A, Ireland K, Legg K, Keane R, Barge E, Jenkins M, et al Complex Challenges of Maintaining Whitebark Pine in Greater Yellowstone under Climate Change: A Call for Innovative Research, Management, and Policy Approaches. Forests. 2016;7: 54.

[55] Lorenz TJ, Sullivan KA. Comparison of survey methods for monitoring Clark’s Nutcrackers and predicting dispersal of whitebark pine seeds: Monitoring Clark’s Nutcrackers. J Field Ornithol. 2010;81: 430–441.

[56] Maier ME. Clark’s Nutcracker Seed Harvest Patterns in Glacier National Park and a Novel Method for Monitoring Whitebark Pine Cones. 2012. http://digitalcommons.usu.edu/etd/1275/.

[57] Greater Yellowstone Whitebark Pine Monitoring Working Group. Monitoring whitebark pine in the Greater Yellowstone Ecosystem: 2018 annual report. Natural Resource Data Series NPS/GRYN/NRDS—2019/1225. National Park Service, Fort Collins, Colorado. 2019.

[58] Rebstock GA, Boersma PD, García-Borboroglu P. Changes in habitat use and nesting density in a declining seabird colony. Popul Ecol. 2016;58: 105–119.

[59] Cohen JB, Houghton LM, Fraser JD. Nesting Density and Reproductive Success of Piping Plovers in Response to Storm- and Human-Created Habitat Changes. Wildl Monogr. 2009; 1–24.

[60] Holling CS. Adaptive Environmental Assessment and Management. Chichester, UK: John Wiley and Sons 1978.

[61] Walters CJ. Adaptive Management of Renewable Resources. New York: Macmillan 1986.

