# Supplementary material for: Landscape- and local-scale habitat influences on occurrence and detection probability of Clark’s nutcrackers: Implications for conservation
Source: PLoS One. 2020 May 29;15(5):e0233726. doi: 10.1371/journal.pone.0233726 (PMC7259736; doi:10.1371/journal.pone.0233726)
Supplement: S1 Text — (PDF) [file pone.0233726.s001.pdf]

Appendix 1: Clark's nutcracker occurrence in a region with whitebark pine decline: implications for conservation of the mutualism

Taza Schaming & Chris Sutherland

June 12, 2016

1 Overview

In this appendix, we provide some additional details about the *final* occupancy model used in this study, i.e., the model used to produce the results presented in the main text. The inference objective here is to model Clark's nutcracker occurrence in each of $s = 1, \ldots, 5$ biological seasons (BR: breeding season, ES: early and LS: late summer, FH: fall seed harvest, and PH: post-harvest). In the $t = 1, \ldots, 5$ years, each of the $i = 1, \ldots, 335$ sites were visited, during which $j = 1, \ldots, 3$ 10-minute detection/non-detection surveys were conducted, and the detection ($y = 1$) or not ($y = 0$) of Clark's nutcrackers was recorded.

2 Observation process - *detectability*

Rarely can a species be detected with absolute certainty and conducting multiple visits to sites in close succession in a period that is assumed to be *closed* provides the 'repeated visit' data required to estimate, and thus

account for, imperfect detection of the true occupancy state.

Preliminary analysis showed no support for any covariate effects on detectability based on the 0.5 posterior model support threshold cut-off recommended by Barbieri and Berger (2004). In the absence of covariates, but to allow for variation in detectability, we modeled year- (t) and season- (s) specific detection probability as a normally distributed random effect with a single (global) mean (μ_θ) and standard deviation (σ_θ):

$$\theta_{s,t} \sim \text{Norm}(\mu_\theta, \sigma_\theta^2).$$

Due to a lack of fit of the detection model (see below), we allowed for additional variation at the level of the unique site-season-year combination also as a normally distributed random effect with a mean of 0 and a standard deviation (σ_ϵ) to be estimated:

$$\epsilon_{i,s,t} \sim \text{Norm}(0, \sigma_\epsilon^2).$$

Binary observations ($y = 1$ when detected and $y = 0$ otherwise) at site i, during survey j, in season s in year t, are then modeled as Bernoulli random variables with success probability p which is multiplied by $z_{i,s,t}$, the latent occurrence state of site i, in season s, in year t, setting detection probability to $p = 0$ when sites are unoccupied and p when occupied:

$$y_{i,j,s,t} \sim \text{Bern}(p_{i,j,s,t} \times z_{i,s,t}),$$

where

$$\text{logit}(p_{i,j,s,t}) = \theta_{s,t} + \epsilon_{i,s,t}.$$

The detection parameters to be estimated, and the (uninformative) priors used in the Bayesian analysis, are provided in Table 1.

Table 1: Detectability parameters and priors used in the analysis of the Clark's nutcracker data.

Parameter	Prior
μ_θ	$Normal(0, 1000)$
σ_θ	$Gamma(0.1, 0.1)$
σ_ϵ	$Gamma(0.1, 0.1)$

3 Ecological process - *occurrence*

The interest is in estimating ψ_{ist}, the probability that site i is occupied in season s in year t, and specifically, to relate these probabilities to site specific covariates. Again, we conducted a preliminary analysis and, using the 0.5 posterior model support threshold cut-off recommended by Barbieri and Berger (2004), selected the final set of covariates which were: whitebark pine cones per hectare (cone crop: cc), the proportion of whitebark pine within 32.6 km radius (pw), and the proportion of Douglas-fir within 3.2 km radius (pd). In addition, and to account for extreme zero-inflation in cone counts, cone crop was included both as both a binary covariate ($cc^I = 1$ if cone count is 0 and $cc^I = 0$ otherwise), and as continuous covariate of observed cone crop counts (cc). This allows the intercept of the occurrence model to vary depending on whether or not cones are present.

We modeled year and season covariate effects as covariate-specific (k) random effects, i.e., with a covariate-specific mean ($\mu_{\beta,k}$) and standard deviation ($\sigma_{\beta,k}$):

$$\beta_{k,s,t} \sim \text{Norm}(\mu_{\beta,k}, \sigma^2_{\beta,k}),$$

for the $k = 5$ parameters of interest, i.e., the intercept, and the four covariate effects (cc, cc^I, pw,and pd).

The resulting *global* model is:

$$logit(\psi_{i,t,s}) = \beta_{1,t,s} + \beta_{2,ts} \times cc_{i,t,s} + \beta_{3,ts} \times cc^{I}_{i,t,s} + \beta_{4,ts} \times pw_{i,t,s} + \beta_{5,ts} \times pd_{i,t,s}$$

We adopted an *cross-product* model selection approach where each plausible model has an equal *prior inclusion probability* which is $1/M$, where M is the number of candidate models. The candidate models for each season were informed by the biological knowledge of the species and the hypotheses of interest (see main text). Cross-product model selection is a model-based version of variable selection which involves the estimation of the M cell probabilities of a categorical distribution. The posterior distribution of these cell probabilities describes the posterior model support for the candidate set of M models. Each category (model) of the categorical distribution represents a vector of binary indicator variable for each term in the global model, i.e., $\boldsymbol{\omega} = \omega_1, \ldots, \omega_5$, which includes or removes the kth term depending on whether $\omega_k = 1$ or $\omega_k = 0$, respectively. Thus, the general model can be reformulated to allow for cross-product model selection as follows:

$$logit(\psi_{i,t,s}) = \beta_{1,t,s} + \omega_{m,1} \times \beta_{2,ts} \times cc_{i,t,s} + \omega_{m,2} \times \beta_{3,ts} \times cc^{I}_{i,t,s} + \omega_{m,3} \times \beta_{4,ts} \times pw_{i,t,s} + \omega_{m,4} \times \beta_{5,ts} \times pd_{i,t,s}$$

Note that the intercept term, β_1, is always included in the model.

There are two sets of priors to be considered: the priors for the *parameters* to be estimated (Table 2), and the season-specific prior model probabilities (Table 3).

Although prior model probabilities were set to be equal, i.e., each model had an equal probability of being selected, there were certain covariate combinations that, based on the biology of Clark's nutcrackers, were considered not plausible *a priori*. Thus, prior model probabilities are equal across *all plausible model*

Table 2: Occurrence effects parameters and priors used in the analysis of the Clark's nutcracker data. WBP = whitebark pine and DF = Douglas-fir.

Parameter	Covariate	Prior
$\mu_{\beta,1}$	Intercept ($mean$)	$Normal(0, 1000)$
$\mu_{\beta,2}$	WBP cone crop count ($mean$)	$Normal(0, 1000)$
$\mu_{\beta,3}$	WBP cone crop absence ($mean$)	$Normal(0, 1000)$
$\mu_{\beta,4}$	Proportion WBP within 32.6km radius ($mean$)	$Normal(0, 1000)$
$\mu_{\beta,5}$	Proportion DF within 3.2km radius ($mean$)	$Normal(0, 1000)$
$\sigma_{\beta,1}$	Intercept (sd)	$Gamma(0.1, 0.1)$
$\sigma_{\beta,2}$	WBP cone crop count (sd)	$Gamma(0.1, 0.1)$
$\sigma_{\beta,3}$	WBP cone crop absence (sd)	$Gamma(0.1, 0.1)$
$\sigma_{\beta,4}$	Proportion WBP within 32.6km radius (sd)	$Gamma(0.1, 0.1)$
$\sigma_{\beta,5}$	Proportion DF within 3.2km radius (sd)	$Gamma(0.1, 0.1)$

for a given season (Table 3).

Table 3: Season-specific candidate models for the occurrence. The table shows both model formulation (• denotes inclusion in a model), and prior model probabilities. Non-zero prior model probabilities represent the *plausible* candidate model set.

	Parameters					Priors				
Model	*Intercept*	*cc*	cc^I	*pw*	*pd*	BR	ES	LS	FH	LH
1	•					0.25	0.50	0.25	0.25	0.25
2	•	•	•			0.00	0.00	0.25	0.25	0.00
3	•			•		0.25	0.50	0.25	0.25	0.25
4	•				•	0.25	0.00	0.00	0.00	0.25
5	•	•	•	•		0.00	0.00	0.25	0.25	0.00
6	•			•	•	0.25	0.00	0.00	0.00	0.25

4 JAGS model code

```
model {

## PRIORS

# detection priors:

  # global: norm(mu,sd)
    mu.p.int ~ dnorm(0,0.01)
    tau.p.int ~ dgamma(0.1,0.1)
    logit(lp.mu) <- mu.p.int
  # random effects
  for(s in 1:S){#season
   for(t in 1:T){#year
      p.Int[t,s] <- p.int[t,s] * surveyed.sxt[s,t]
      p.int[t,s] ~ dnorm(mu.p.int, tau.p.int)
  }}
  tau.eps.p ~ dgamma(0.1,0.1)

# occupancy priors:

  mu.psi.int ~ dnorm(0,0.1)
  tau.psi.int ~ dgamma(0.1,0.1)

  # choose model:
  for(s in 1:S){
    g.psi[s] ~ dcat(psiModPriors[,s])
  }

  # fixed effects
  for(cv in 1:5){ #beta
   for(s in 1:S){#season
     psi.beta[cv,s] ~ dnorm(0,0.1)
     psi.Beta[cv,s] <- psi.beta[cv,s] * psiModMat[g.psi[s],cv]
  }}
```

```
## OCCUPANCY MODEL

  for(r in 1:R){#site

    # occupancy for each season
    logit(psi[r]) <- psi.Beta[1, seas[r]]  +
                     psi.Beta[2, seas[r]]  * ccSc[r]  +
                     psi.Beta[3, seas[r]]  * pwSc[r]  +
                     psi.Beta[4, seas[r]]  * pdSc[r]  +
                     psi.Beta[5, seas[r]]  * ccI0[r]
    z[r] ~ dbern(psi[r])

    # observation model
    eps.p[r] ~ dnorm(0, tau.eps.p)
    for(j in 1:J){#visit
      logit(lp[r,j]) <- p.Int[year[r],seas[r]] + eps.p[r]
      y[r,j] ~ dbern(lp[r,j] * z[r])     #Observed
      y.new[r,j] ~ dbern(lp[r,j] * z[r]) #Expected
    }
  }

  # goodness-of-fit
  for(r in 1:R){#site
    exp[r,1] <- (1-y.new[r,1])* (1-y.new[r,2])* (1-y.new[r,3]) #000
    obs[r,1] <- (1-y[r,1])    * (1-y[r,2])    * (1-y[r,3])     #000
    exp[r,2] <- (y.new[r,1])  * (1-y.new[r,2])* (1-y.new[r,3]) #100
    obs[r,2] <- (y[r,1])      * (1-y[r,2])    * (1-y[r,3])     #100
    exp[r,3] <- (1-y.new[r,1])* (y.new[r,2])  * (1-y.new[r,3]) #010
    obs[r,3] <- (1-y[r,1])    * (y[r,2])      * (1-y[r,3])     #010
    exp[r,4] <- (1-y.new[r,1])* (1-y.new[r,2])* (y.new[r,3])   #001
    obs[r,4] <- (1-y[r,1])    * (1-y[r,2])    * (y[r,3])       #001
    exp[r,5] <- (y.new[r,1])  * (y.new[r,2])  * (1-y.new[r,3]) #110
    obs[r,5] <- (y[r,1])      * (y[r,2])      * (1-y[r,3])     #110
    exp[r,6] <- (y.new[r,1])  * (1-y.new[r,2])* (y.new[r,3])   #101
    obs[r,6] <- (y[r,1])      * (1-y[r,2])    * (y[r,3])       #101
    exp[r,7] <- (1-y.new[r,1])* (y.new[r,2])  * (y.new[r,3])   #011
    obs[r,7] <- (1-y[r,1])    * (y[r,2])      * (y[r,3])       #011
```

```
    exp[r,8] <- (y.new[r,1])  * (y.new[r,2])  * (y.new[r,3])   #111
    obs[r,8] <- (y[r,1])      * (y[r,2])      * (y[r,3])       #111
  }
  for(i in 1:8){
    a[i] <- sum(obs[1:R,i])
    b[i] <- sum(exp[1:R,i])
  }
} # end of model
```

5 Model Fitting

To fit these models, we use `JAGS` (Plummer 2003), called from within `R` (R Core Development Team) using the library `jagsUI` (Kellner 2016). The data are available as an `R` workspace available as the supplement (`nutcracker.RData`). Contained in this workspace are two data objects:

- 100m radius data `nutData.100`
- infinite radius data `nutData.all`

Within each object, all the data required to each model is contained:

- the detection/non-detection data, `y`
- the covariates (see Table 1)
- the dimensions variables for the number of rows in the data, `R`
- the number of visits, `J`
- the number of seasons, `S`
- the number of years, `T`
- the season of the site visit, `seas`
- the year of the site visit, `year`
- the occurrence model matrix, `psiModMat`
- the associated model prior probabilities, `psiModPriors`
- a binary matrix indicating which season-year combinations surveys were

conducted, `surveyed.sxt`

- the mean and standard deviation of each covariate used for scaling, `scale.df`

The data can be loaded:

```
#point the working directory to folder containing nutcracker.RData
setwd(<your.dir>)
load("nutcracker.RData")
```

and inspected:

```
# 100m raduis
names(nutData.100)
```

```
##  [1] "y"            "cc"           "ccI"          "pw"           "pd"
##  [6] "R"            "J"            "S"            "T"            "seas"
## [11] "year"         "psiModMat"    "psiModPriors" "surveyed.sxt" "scale.df"
```

```
# all distances
names(nutData.all)
```

```
##  [1] "y"            "cc"           "ccI"          "pw"           "pd"
##  [6] "R"            "J"            "S"            "T"            "seas"
## [11] "year"         "psiModMat"    "psiModPriors" "surveyed.sxt" "scale.df"
```

From these data an additional data object must be created to pass to `JAGS` for fitting the models:

```
#100m radius data
Jdata.100 <- list(y = nutData.100$y, cc = nutData.100$cc,
                  ccI = nutData.100$ccI, pw = nutData.100$pw,
                  pd = nutData.100$pd, seas = nutData.100$seas,
                  year = nutData.100$year, R=nutData.100$R,
                  J = nutData.100$J, S = nutData.100$S,
```

```
                   T = nutData.100$T, surveyed.sxt = nutData.100$surveyed.sxt,
                   psiModMat = nutData.100$psiModMat,
                   psiModPriors = nutData.100$psiModPriors)

#All distance data
Jdata.all <- list(y = nutData.all$y, cc = nutData.all$cc,
                   ccI = nutData.all$ccI, pw = nutData.all$pw,
                   pd = nutData.all$pd, seas = nutData.all$seas,
                   year = nutData.all$year, R=nutData.all$R,
                   J = nutData.all$J, S = nutData.all$S,
                   T = nutData.all$T, surveyed.sxt = nutData.all$surveyed.sxt,
                   psiModMat = nutData.all$psiModMat,
                   psiModPriors = nutData.all$psiModPriors)
```

It is preferable to create a function that generates reasonable starting values to initialize the model. Initial values are required for the parameters that are to estimated. In addition, we specify initial values for the latent occupancy states z.

```
#Create initial values function
zst <- rep(1,length(nutData.100$y))
inits <- function(){
  list(mu.p.beta = runif(1,-1,1),
       sd.p.beta = runif(1,0,2),
       mu.psi.beta = runif(5,-1,1),
       sd.psi.beta = runif(5,0,2),
       z = zst
       )}
```

Next, we define the MCMC settings and define the parameters for which posterior summaries are required:

```r
#Jags settings
n.chains  <-      3 #number of chains
n.adapt   <-   5000 #length of adaption phase
n.burnin  <-  50000 #number of itereations discarded as burnin
n.iter    <- 100000 #number of samples/iterations
n.thin    <-      1 #thinning rate
params <- c("g.p","g.psi","p.Beta","psi.Beta") #parameters of interest
```

And finally, with the data constructed and MCMC setting defined, the models can be fit. As with the data objects, the `JAGS` model file is provided as a supplement (`nutcrackerBugs.txt`).

```r
library("jagsUI")
# create an object called 'wd' which is the path of the folder
# where the 'nutcrackerBugs.txt' file is located:
wd <- "<your.dir>"

#Fit the 100m model
out.100 <- jags(model.file = paste0(wd,"nutcrackerBugs.txt"),
                data = Jdata.100,
                parameters.to.save = params,
                n.chains = n.chains,
                n.adapt = n.adapt,
                n.burnin = n.burnin,
                n.iter = n.iter,
                n.thin = n.thin,
                inits = inits)

#Fit the all distance model
out.all <- jags(model.file = paste0(wd,"nutcrackerBugs.txt"),
                data = Jdata.all,
```

```
                    parameters.to.save = params,
                    n.chains = n.chains,
                    n.adapt = n.adapt,
                    n.burnin = n.burnin,
                    n.iter = n.iter,
                    n.thin = n.thin,
                    inits = inits)
```

The output of both models have been supplied as supplements and can be accessed as follows:

```
#point the working directory to folder containing output .RData files
setwd(<your.dir>)
load("out.100.RData")
load("out.all.RData")
out.100
out.all
```

6 Goodness of fit

Below is the code for computing the posterior distribution of the Pearson's chi-square statistic comparing the *observed* (y) and *expected* encounter history frequencies (see `JAGS` code above).

```
#GOF for the 100m model
par(mfrow=c(1,2), oma=c(0,0,0,0),mar=c(4,4,1,1))
freq.exp <- out.100$sims.list$b
freq.obs <- out.100$sims.list$a
d.f <- 7
fit.100 <- apply((freq.obs - freq.exp)^2/freq.exp,1,sum)-qchisq(0.95,d.f)
hist(fit.100, main = "Distance: 100m",probability = T,
```

```r
     xlim=c(-75,75),ylim=c(0,0.075), las=1,
     xlab="Obs - Exp")
abline(v=0,lwd=2,col=4); box(bty="l")

#GOF for the all-distance model
freq.exp <- out.all$sims.list$b
freq.obs <- out.all$sims.list$a
d.f <- 7
fit.all <- apply((freq.obs - freq.exp)^2/freq.exp,1,sum)-qchisq(0.95,d.f)
hist(fit.all, main = "Distance: all",probability = T,
     xlim=c(-75,75),ylim=c(0,0.075), las=1,
     xlab="Obs - Exp")
abline(v=0,lwd=2,col=4); box(bty="l")
```

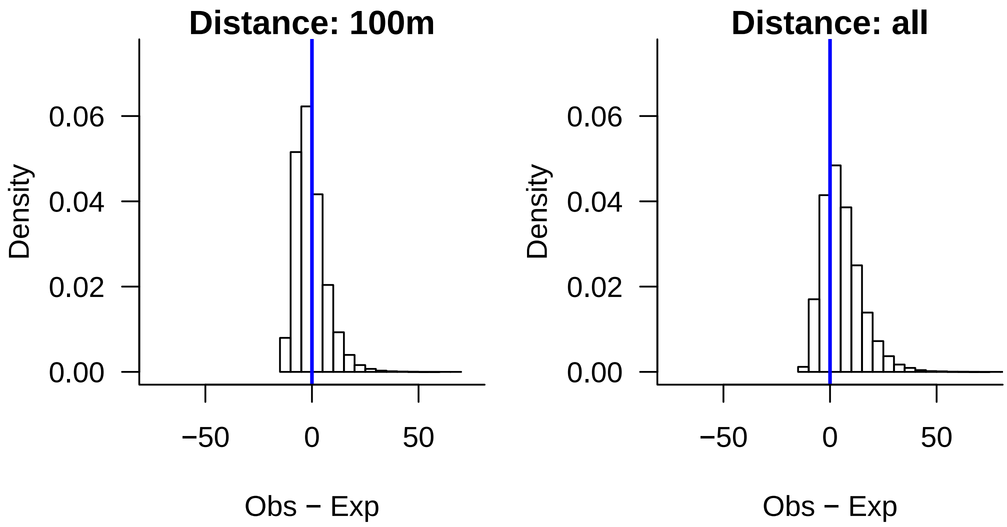


```r
print(mean(fit.100>0))
```

```
## [1] 0.3909667
```

```r
print(mean(fit.all>0))
```

```
## [1] 0.7016333
```

7 References

Barbieri, M.M. and Berger, J.O. (2004). Optimal predictive model selection. Annals of Statistics, pp.870-897.

Kellner, K. (2016). jagsUI: A Wrapper Around 'rjags' to Streamline 'JAGS' Analyses. R package version 1.4.2. https://CRAN.R-project.org/package=jagsUI.

R Core Team (2016). R: A language and environment for statistical computing. R Foundation for Statistical Computing, Vienna, Austria. URL https://www.R-project.org/.
