# Supplementary material for: Landscape- and local-scale habitat influences on occurrence and detection probability of Clark’s nutcrackers: Implications for conservation
Source: PLoS One. 2020 May 29;15(5):e0233726. doi: 10.1371/journal.pone.0233726 (PMC7259736; doi:10.1371/journal.pone.0233726)
Supplement: S2 Text — (PDF) [file pone.0233726.s002.pdf]

Appendix 2: Variation in study design between this study, McKinney et al. (2009) and Barringer et al. (2012).

The sample effort varied considerably between studies, with a lower sample effort in previous research (number of survey points, number of surveys, total number of hours of surveys, number of years of surveys in each region; Table 1). Because nutcracker occurrence can be highly variable, the higher sampling effort in this study would have better assessed the Clark's nutcracker-whitebark pine relationship in the study area. On the other hand, the inference from comparing Clark's nutcracker behavior between multiple ecosystems, as done by McKinney et al. [1] and Barringer et al. [2], contributes to a better understanding of how these birds are impacted by habitat metrics in geographically distinct habitats that vary widely in whitebark pine abundance and health, alternative food sources, and climate, among other variables.

Table 1. A comparison of this study, McKinney et al. (2009) and Barringer et al. (2012).

**Study**	This study	McKinney et al. [19]	Barringer et al. [20]
**Detectability assessed?**	Yes	No	No
**Total number of years**	5 (2009 – 2013, but few surveys in 2010)	5 (2 – 3 years/region; 2001 – 2002, 2004 - 2006)	2 (2008 – 2009)
**Total number of survey regions**	1 (GYE)*	3 (GYE, ND, BM)*	2 (GYE, ND)*
**Total number of survey points**	238 (n = 48: 8/transect, 8 1 km transects; n = 190: random points, ≥400 km apart) [22]	24 sites (site = 100 m x ≥200 m (2 - 7 ha, mean = 2.6 ha), 2 - 4 1 ha squares sampled/site)	60 (6/transect, 10 1 km transects)
**Total number of surveys**	3,135 (3 counts/ visit)	42 site-years	480 (2x/day, 2 visits/yr)

**Total survey hours**	522.5 (late summer = 166.5, fall = 138**)	84 – 168 assumed, since 2 - 4 ha surveyed/site; 1h/1ha, but total observation hours unclear	80
**Duration of surveys**	10 min counts (3 sequential counts = 30 min at point/visit)	1ha/1hr; 2 -4 hrs/site	10 min counts
**Survey dates**	Mar 15 – Oct 1 (divided into 5 stages)	Jun 29 – Sept 6	mid-late July, late Aug - early Sept
**Late summer and fall surveys separated or combined in analyses**	separated	combined	combined
**Survey radius**	100 m and infinite (analyzed separately)	1 ha surveys (2 – 4 ha/site)	infinite
**Habitat assessment**	At each point (n = 238): 1,900 m^2 of belt transects, and 5 point quarters	1,000 m^2 within each 1 ha square (n = 24 sites, subdivided into 2 – 4 ha squares)	2 habitat surveys, totaling 1,000 m^2 , at randomly selected 100 m sections along each transect (all 6 points on a transect considered to have the same habitat)
**Cone crop predictor variable**	Continuous and binary (presence/ absence)	Continuous	Continuous
**Landscape scale habitat variables**	Included (whitebark pine within 32.6 km, Douglas-fir within 3.2 km)	None included	None included

* GYE = Greater Yellowstone Ecosystem, ND = Northern Divide Ecosystem, BM = Bitterroot Mountains

Effort in assessing habitat also varied considerably (Table 1). We observed that whitebark pine regularly grows in a habitat mosaic with other conifer species, often interspersed with open patches, rather than in homogenous stands. Within a stand, we also regularly observed high variation in whitebark pine age class, density, and health, often rapidly changing multiple times within a few hundred meters. During our initial 2009 point counts, we originally only conducted two belt transects per 1 km transect, but quickly observed that these transects were not adequately capturing habitat at the point. We therefore added additional belt transects and modified point quarter surveys at every point to more accurately assess habitat.

We also separately analyzed then compared data from 100 m and infinite radius, finding some results varied in significance depending on the survey radius. McKinney et al. [19] surveyed birds within 1 ha (100 m x 100 m), and Barringer et al. [20] included all nutcracker observations, using an infinite radius.

References

1. McKinney ST, Fiedler CE, Tomback DF. Invasive pathogen threatens bird-pine mutualism: implications for sustaining a high-elevation ecosystem. Ecological Applications. 2009;19: 597–607.
2. Barringer LE, Tomback DF, Wunder MB, McKinney ST. Whitebark Pine Stand Condition, Tree Abundance, and Cone Production as Predictors of Visitation by Clark's Nutcracker. Newsom LA, editor. PLoS ONE. 2012;7: e37663.
